# A Dean's Opinion

**Published:** 1889-09-14

**Authors:** 


### A Dean's Opinion.

AN INTERVIEW WITH MR. PEARCE GOULD.

"Our students," said Mr. Pearce Gould, in answer to the first question put to him by our commissioner, "our students are of all ages from sixteen to about forty. Sixteen sounds rather young, I admit, but age is hardly to be tested by years; much depends on the man himself and the influences of his home life and education. The average age at which a youth begins medical study is, I should say—speaking not from statistics, but from the impression given by the men's aspect and conversation—about eighteen. That is a very good age; on the whole it is better that a man should begin to learn his profession young. A few go through a university course before they take up medicine, and these, of course are older. That they are better does not inevitably follow. University education is no drawback in itself, but it implies the possession of a certain amount of wealth; sometimes too much, I fear, to be conducive to industry. Poverty is a hard thing for a man to struggle with; it brings disabilities enough; but, perhaps, wealth is still more difficult to contend with.

"Yet on the side of the university training is to be ranked the possibility it gives of sending the medical student to his curriculum with a better education than most of them possess. Medical students are, as a rule, deficient in breadth of education. You can discover that from their conversation; it is often all 'shop.' Interest in sports is sometimes the only variety to the monotonous talk about cases and classes. You will find a few musical men in all schools, and so far that is good, but of general culture, or even interest in things outside the professional work, there is little or none. The majority of students cannot speak or read French or German, or any foreign language. Then they come to us almost wholly ignorant of science. A few may have a smattering of chemistry, but nothing worth speaking of. Most have only the very slight knowledge of mechanics necessary to scrape through the preliminary examination, no more. The result of this is that valuable time has to be spent during the curriculum in teaching things that should have been acquired before. Think of the time that might be saved in a physiology class if the men only understood the main facts of statics, dynamics, hydrostatics, optic, acoustics, and electricity.

"This waste of time in teaching facts that should not be left for the period of medical study, is the more to be regretted that the time itself is so short. Four years in which to teach the whole of medicine and surgery! It is not enough, and our men leave just when they are best fitted to profit by their studies. A few remain for a fifth year as resident physicians or surgeons in the hospital, but with many this is impossible. They have to earn their living, and their fathers think, naturally, that enough time has been spent in preparing them for their life work. But if the time is brief, it is the more necessary that all of it should be available for professional study. Of course, we have some men whose study is prolonged beyond the necessary four years for very unsatisfactory reasons. There are idlers in every community, and it is hardly remarkable that very young men, plunged into the liberty of London life, should occasionally drift into evil ways. It is the aim of a dean of a school to stir up these idlers before they have acquired a confirmed habit of neglecting their work. Every attendance is registered, and unless a man attends two-thirds of the lectures in each course, and goes up for all the class examinations, he does not receive his certificate of attendance. Moreover, a report is also sent to his parents or guardians twice a year, so that they may really know how he goes on. That, at least, is our rule, but a little discretion is required in acting upon it. Sometimes in a thoughtless but good-hearted lad a few words of warning, and the knowledge that he is under a friendly but constant supervision, do all that is required. When a man is incorrigibly idle we have him removed, but before doing so we generally give him a last chance. He is told that unless he passes an examination within a certain given time, he must go, and it is surprising how well this works. A man, taken aback by this warning, pulls himself together, and not only passes his examination, but acquires a habit of industry, which is a much more important matter. I have known men who would otherwise have gone to ruin saved by being thus taken in time.

"Of course, the supervision I speak of is possible only in a school of moderate size. Where the new entries do not exceed fifty or sixty each year, it can be managed, and thus I would give the preference, for the student's sake, to a school that is neither very large nor very small. In the latter there is too little of the spirit of emulation to animate the student to do his best, and also—a point which cannot be ignored—a small school cannot hope to keep a first-class staff of teachers. If clever young men go there, they do so merely as a step to something higher. On the other hand, in a large school the student becomes lost in the crowd. He may go from bad to worse without anybody knowing it. Still, for really first-rate men, the men who would do their work well anywhere, there is nothing like the inspiration that comes by the rivalry in a large college. Perhaps, however, I ought to limit my approval to the first part of the course, where attendance on lectures is the chief duty. When it comes to clinical work, a large school—by this I mean a school large in proportion to the hospital attached to it—has a decided disadvantage. The student has not enough of that personal charge of patients which is essential to success in practice. When only the best students get dresserships and clerkships, what is to become of the rest? We make it a rule that all our men should, at one time or another, hold all the non-resident clinical posts at the hospital. They begin in the out-patient room. There they are simultaneously dresser to a surgeon, assistant to a dental surgeon, and assistant to an oculist. After holding these posts three months they become clerk to a physician, and assist in the ear and throat department, and in gynæcology. Then they enter the wards and are dressers and clinical clerks for six months each, and as such they have each from six to fifteen patients under their charge. Thus every man who goes through the curriculum has seen something of every department of medicine and surgery, and has worked practically at it. It is necessary to see to this, for when any department—eye, ear, or skin diseases, for example—is removed from the general wards it is apt to be neglected. A man may thus go out into the world ignorant of many things in his profession, and from the imperfect education of the general practitioner arises the demand for the specialist. A great deal is done by making the examinations thorough, "all-round," and searching; but, besides this, it is necessary to see that every man has a practical acquaintance with his work enforced on him as far as possible.

"In thus speaking of making the student learn, I may seem to imply that students, as a class, are idle. I do not mean this, but only that they are apt to confine their studies to somewhat narrow limits. But they are, in general, a hard-working, industrious class. I know men who work from nine in the morning till eleven at night, without any intermission beyond the necessary time for meals, and indeed, with the amount they have to learn during their four years, they cannot be other than studious. In the last place, I wish to say that they are, on the whole, moral men. I should not need to say that they are not rowdy, but I know that the old mythical portrait of the medical student still exists in the popular imagination; whatever truth was in it has long passed away. The whole tone of the medical profession has risen immensely during the last twenty years, and students have been affected by the change as much as others. Among other things, the presence of ladies in hospitals has exercised a wholesome influence. I do not wish to over-praise our students, or to pretend that they are all that they ought to be, though the influence of the honest, manly Christians among them is great and genuine. Poverty often throws them into very bad company, the lack of culture I spoke of sometimes predisposes them to low amusements, and the temptations that surround young men suddenly endowed with complete independence are no doubt great; but, taking them as a whole, their record will compare favourably with that of any other class of the community, and is such as to make them worthy of the profession they have taken up."

